# Prediction of Side Effects Using Comprehensive Similarity Measures

**DOI:** 10.1155/2020/1357630

**Published:** 2020-02-27

**Authors:** Sukyung Seo, Taekeon Lee, Mi-hyun Kim, Youngmi Yoon

**Affiliations:** ^1^Department of Computer Engineering, Gachon University, Seongnam, Republic of Korea; ^2^Gachon Institute of Pharmaceutical Science and Department of Pharmacy, College of Pharmacy, Gachon University, Yeonsu-gu, Incheon, Republic of Korea

## Abstract

Identifying the potential side effects of drugs is crucial in clinical trials in the pharmaceutical industry. The existing side effect prediction methods mainly focus on the chemical and biological properties of drugs. This study proposes a method that uses diverse information such as drug-drug interactions from DrugBank, drug-drug interactions from network, single nucleotide polymorphisms, and side effect anatomical hierarchy as well as chemical structures, indications, and targets. The proposed method is based on the assumption that properties used in drug repositioning studies could be utilized to predict side effects because the phenotypic expression of a side effect is similar to that of the disease. The prediction results using the proposed method showed a 3.5% improvement in the area under the curve (AUC) over that obtained when only chemical, indication, and target features were used. The random forest model delivered outstanding results for all combinations of feature types. Finally, after identifying candidate side effects of drugs using the proposed method, the following four popular drugs were discussed: (1) dasatinib, (2) sitagliptin, (3) vorinostat, and (4) clonidine.

## 1. Introduction

Research and development efforts in the pharmaceutical industry have been low during the past few decades [1]. Most drug candidates, owing to their side effects, fail to receive the approval of the US Food and Drug Administration (FDA) for their commercialization. Identifying the undesirable off-target activities of potential drugs, capable of causing side effects and, in turn, resulting in drug discovery failures, is a challenge in the drug-development process. While most of the serious side effects are identified during preclinical and clinical trials, some of them are reported during the postapproval monitoring. The uncertainty about the potential side effects of new drugs is a concern for not only pharmaceutical companies but also patients because they pose a health risk and can even cause death [2].

The existing computational methods used to predict the side effects of drugs assume that similar drugs have comparable properties in terms of chemical and biological characteristics, such as their structures and targets. Pauwels et al. predicted potential drug side effects using a sparse canonical correlation analysis model based on chemical structures, whereas Mizutani et al. developed a method based on chemical structures of drugs and target proteins [3, 4]. It has been recognized that drugs with similar chemical structures exhibit comparable biological activities [5]. The screening of a significant number of chemical databases containing the structures of available chemicals is a pivotal process even in drug design studies that predict the properties of chemical compounds [6]. It is logical to expect that common drug targets that trigger similar therapeutic effects induce similar signaling cascades and, therefore, similar side effects.

Previous research on side effect prediction was extended to phenotypic traits after much focus on chemical and biological properties. Liu et al. integrated drug-phenotypic information, besides chemical and biological information, into the features for machine learning and demonstrated significant improvements in the prediction results [7]. Zheng et al. utilized therapeutic data in addition to drug substitutes, chemical structures, and targets. These two studies were based on the idea that drugs with similar therapeutic effects may have comparable side effects [8].

However, a vast majority of previously conducted studies did not fully utilize the existing knowledge on drugs. This study focuses on the idea that the phenotypic expression of a side effect can be similar to that of a disease. Furthermore, because drugs are subject to complex influences such as metabolic transformations and other pharmacokinetic transformations while they are metabolized and physiologically distributed, their side effects cannot be simply predicted by their chemical properties [9]. Thus, it was assumed that similarities at a molecular level used in drug repositioning can be applied to the prediction of side effects; moreover, various similarity measurements could be helpful in improving the predictive capabilities of the model. Single nucleotide polymorphisms (SNPs) and drug-drug interactions (DDIs) that were not used by previous side effect prediction studies were utilized because both have been used in drug repositioning studies, where they have demonstrated outstanding results [10, 11].

This study proposes a machine learning approach for the identification of potential drug side effects by leveraging various information resources on drug and side effect properties, such as (1) drug-drug interactions from DrugBank (DDIs-D), (2) drug-drug interactions from network (DDIs-N), (3) SNPs, (4) chemical structures, (5) indications, (6) targets, and (7) side effect anatomical hierarchy. A formulated set of seven features and diverse machine learning algorithms were adopted to develop a drug-side effect pair. The results showed that using new features proposed in this study in addition to chemical, indication, and target features improved the predictive capability of the side effect prediction model. Furthermore, optimizing the machine learning model to achieve maximal prediction performance resulted in the identification of unlabeled side effects of approved drugs. The following four popular drugs are discussed in the study: (1) dasatinib, (2) sitagliptin, (3) vorinostat, and (4) clonidine.

## 2. Materials and Methods

In this study, the side effects of drugs were predicted using diverse properties, such as (1) DDIs-D, (2) DDIs-N, (3) SNPs, (4) chemical structures, (5) indications, (6) targets, and (7) side effect anatomical hierarchy. Based on these properties, similarities used for feature values were calculated. Next, a dataset was developed in which a drug-side effect pair was a sample, and feature values were assigned based on the known associations from the training samples. Finally, machine learning algorithms were applied to these datasets, and the evaluation results were obtained.

**Figure 1:**
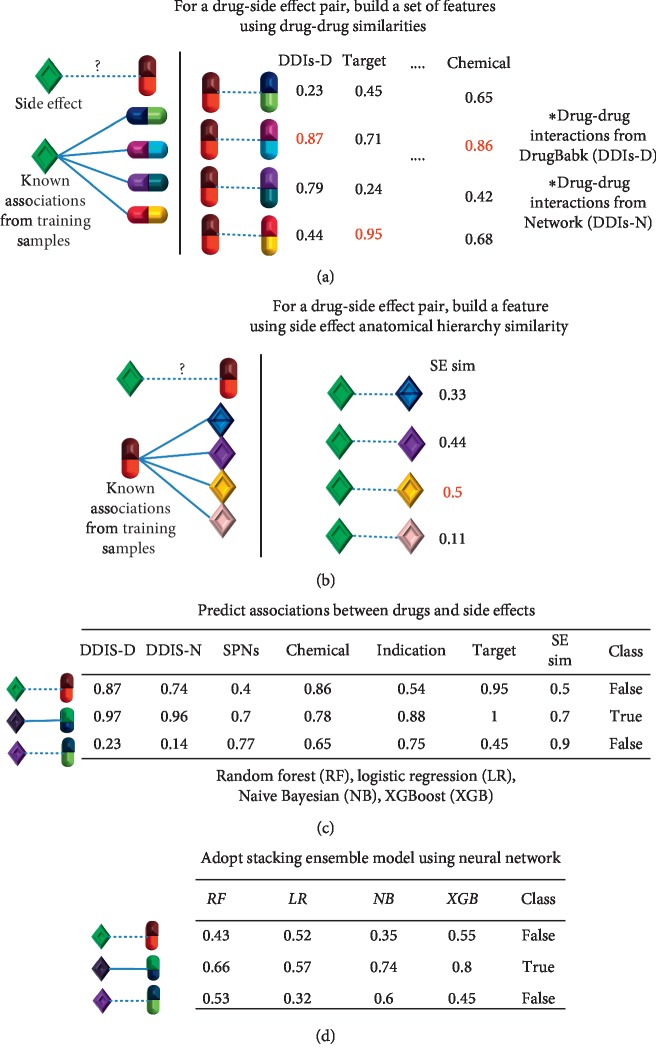
System overview. (a) To build a set of features for a drug-side effect pair, the maximum similarity was selected for each feature based on the known associations from the training samples between the side effect and other drugs. (b) At this step, the maximum side effect anatomical hierarchy similarity was chosen based on the known side effects of the drug from the training samples. (c) By assigning values, as was done for (a) and (b), datasets for machine learning were created with different combinations of features, and diverse classification algorithms, including a random forest, XGBoost, logistic regression, and naive Bayesian model, were applied to predict the relationship between a side effect and a drug. (d) Stacking ensemble learning that incorporated all four classifiers as its base classifiers and used a neural network as its meta classifier was applied.

Figure 1 provides an overview of the system and a brief description of the method.

### 2.1. Measuring Similarities

A variety of similarities were calculated for the following properties: (1) DDIs-D, (2) DDIs-N, (3) SNPs, (4) chemical structures, (5) indications, (6) targets, and (7) side effect anatomical hierarchy. These were used to indicate that similar drugs for each feature have comparable side effects, as shown in Table 1.

#### 2.1.1. Drug-Drug Interaction

DDI relationships were considered based on the assumption that similar drugs are prone to exhibit comparable reactions to other drugs [14, 15]. The gold standard dataset was acquired from Ryu et al., who compiled DDIs from DrugBank [16, 17]. This dataset comprised 192,284 DDIs with 1,710 different drugs and 86 interaction types. According to Ryu et al., one drug may “affect” or “be affected by” another drug. Interactions between two drugs were expressed using the following notation: (*x, y, z*), where *x* represents the other drug within the interaction, *y* denotes the interaction type, and *z* represents whether the drug “affects” or “is affected by” another drug in the interaction. For example, if drug A was affected by drug B with interaction type 1, the relationship is expressed as (*B*, 1, ←); if drug A affected drug *C* with interaction type 2, the relationship is expressed as (*C*,  2, ⟶). Accordingly, a set of elements of drug A was established (*S*_A_ = { (*B*, 1, ←),  (*C*,  2, ⟶)…}). The same procedure was repeated for all the drugs. The DDIs-D similarity was evaluated considering related drugs and their interaction types.

Additionally, by considering network characteristics, the biological functions of DDIs were analyzed. Because proteins interact with other proteins, drug targets in the human protein interactome were considered. The human PPI network was retrieved from STRING (Search Tool for the Retrieval of Interacting Genes/Proteins); it included 11,754,195 pairs of 10,342 different proteins [18]. For each drug, the component genes of a target-target network were obtained using the shortest paths for targets on the PPI network. Subsequently, the gene ontology terms were obtained. The drug-drug similarity of DDIs-N was evaluated using these terms. The DDIs-N similarity represents the functional similarity between the two drugs.

#### 2.1.2. Single Nucleotide Polymorphism

It is widely accepted that two drugs with similar indications (i.e., diseases) are likely to exhibit comparable side effects. Furthermore, a previous study demonstrated that similarities in SNPs could be leveraged to identify drug repositioning [11]. Thus, genes that are affected by SNPs were used to consider the genetic traits of diseases that were affiliated with two drugs. First, disease-associated SNPs from the DisGeNET were collected [19]. Next, the SNPs were mapped to each drug based on indications. Finally, the SNPs were linked to other genes that were regulated by SNPs for each drug using data from Fagny et al. [12]. These data contained expression quantitative trait loci linking SNPs to tissue-specific regulation of gene transcripts from Genotype-Tissue Expression. The SNP similarity was used to show that the more similar the genetic traits of diseases that were related to two drugs, the more comparable the side effects.

#### 2.1.3. Indication

In this section, drug indications from three different databases were collected to present phenotypic traits. First, the Therapeutic Target Database (TTD), containing 44,481 associations spanning 1,000 drugs and 1,298 diseases, was used [20]. Second, the Comparative Toxicogenomics Database (CTD), consisting of curated and inferred chemical-disease associations, was used [21]. However, among the curated associations, only those that exhibited direct evidence of a chemical-disease association (marked “marker/mechanism” or “therapeutic”) were included, and the inferred associations were excluded. As a result, 150,175 associations, spanning 1,510 drugs and 5,709 diseases, were processed from the CTD. Third, the repoDB contained 6,677 associations spanning 1,519 drugs and 1,229 diseases [22]. Finally, this study comprised 197,547 associations spanning 2,144 drugs with DrugBank ID and 6,556 diseases with UMLS ID. The indication similarity was used to denote the phenotypic traits of drugs.

#### 2.1.4. Targets and Chemical Structures

In this study, 6,076 drug-target associations for 1,366 drugs were obtained from DrugBank. When drugs were related to the same protein, they underwent similar biological processes [8]. Thus, drugs that bind to the same target are associated with the same side effect. The biological characteristics of the drugs were considered based on the target similarity.

The simplified molecular-input line-entry system (SMILES) refers to simplified chemical structures and encoded molecular graphs as a human readable string [23]. We obtained the chemical structures of 1,878 drugs in the SMILES format from PubChem and DrugBank [24]. Chemical properties of drugs play an important role. Furthermore, drugs with similar chemical structures traditionally exhibit comparable biological activities [5]. The Open Babel software was used to evaluate the similarity of all drug pairs based on the Tanimoto coefficient, which is the number of bits in common divided by the union of the bits using drug fingerprints [25].

#### 2.1.5. Jaccard Coefficient

The Jaccard coefficient was used to compute the similarities for all the properties mentioned earlier, except the chemical structures. It is equal to the number of traits in common divided by the union of the traits and is expressed as

(1) $$\begin{array}{c} J\left(D_{\text{A}},D_{\text{b}}\right)=\frac{\left|S_{\text{A}}\;\cap \;S_{\text{B}}\;\right|}{\left|S_{\text{A}}\;\cap \;S_{\text{B}}\;\right|}, \end{array}$$

where SA and SB represent the sets of properties of drug A and drug B, respectively. Five similarities were obtained using the Jaccard coefficient for the following properties: (1) DDIs-D, (2) DDIs-N, (3) SNPs, (4) indications, and (5) targets.

#### 2.1.6. Side Effect Anatomical Hierarchy Similarity

**Figure 2:**
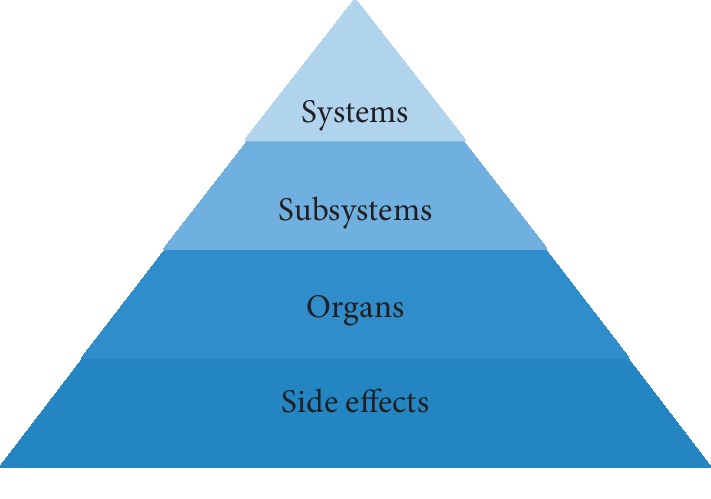
Schema of side effect anatomical hierarchy.

The “side effect anatomical hierarchy” similarity was evaluated using side effect anatomical hierarchy from Wadhaw et al.'s paper [13]. They categorized each side effect manually according to its anatomical scheme, which is organized at three levels: organ, subsystem, and system levels, as depicted in Figure 2. The “side effect” level considers each side effect as an independent side effect. The “organ” level represents organ classes in which side effects are aggregated based on their anatomical schema. The “subsystem” and “system”' levels are aggregated in the same way. Their study implied that similar side effects are likely to be associated with similar anatomical levels. Thus, we named this as “side effect anatomical hierarchy” in our study. Finally, the anatomical hierarchies of 5,868 side effects were obtained.

Using equation (1), the anatomical hierarchies of side effects *a* (SE_*a*_) and *b* (SE_*b*_) for each level were calculated. *S*_*l*_(SE_*a*_,  SE_*b*_) denotes the similarity of two side effects in a specific level and *l* indicates the level.

(2) $$\begin{array}{c} S_{\text{side effect}}\left({\text{SE}}_{a},{\text{SE}}_{b}\right)=\;\frac{\sum_{l=1}^{n}S_{l}\left({\text{SE}}_{a},{\text{SE}}_{b}\right)}{n}. \end{array}$$

In equation (2), *n* denotes the total number of levels, which is 3 in this case. Accordingly, we obtained the anatomical hierarchy similarity *S*_side effect_(SE_*a*_, SE_*b*_) between two side effects.

### 2.2. Assign Similarity Values for Each Drug-Side Effect Sample

In the study dataset, a drug-side effect pair is a sample. To build effective prediction models, only side effects that were related to at least five drugs were considered. In addition, a similarity value between the drug in the pair and other drugs that had the side effect in the pair was required. Positive samples consisting of the known drug-side effect associations from SIDER were generated using the drugs and side effects that met the above condition. In total, 107,878 drug-side effect associations were collected from the SIDER database (reported up to September 2017) [26]. For each drug, the PubChem compound ID was mapped to the DrugBank ID using the annotations provided in biodb.jp [27].

Negative samples were randomly generated from the drugs and side effects used in the positive samples by excluding the known associations. An identical number of drug-side effect samples were collected for the negative and positive sets. Then, a dataset comprising negative and positive sets was built. To avoid a negative sample bias, 100 negative sets were extracted using random sampling with replacement. We performed hold-out validation instead of cross validation because the values in each dataset were highly affected by the positive pairs of the training set. These pairs were split into three subsets: training dataset, validation dataset, and test dataset. The ratio of the three sets was 8 : 1 : 1.

**Figure 3:**
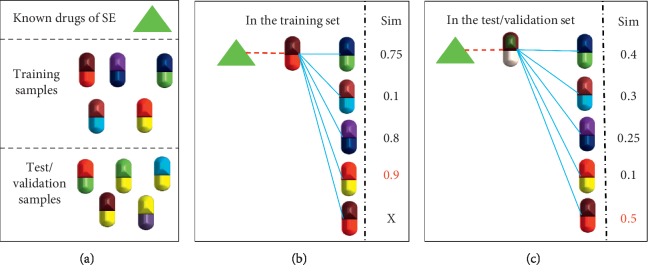
Assigning values to drug-side effect pairs.

Subsequently, datasets were built using the similarity values generated in the above step. First, for each drug-side effect sample, drugs that had known associations with the side effect in the training set were identified. Then, the maximum similarity values for each feature were obtained from the drug and other drugs. In particular, if the drug had a known association with the side effect in the training set, the similarity of the drug itself was excluded. The feature values for the six types of similarities (viz., DDIs-D, DDIs-N, SNPs, indications, targets, and chemical structures) were assigned in the same manner as mentioned above. This step is illustrated in Figure 3.

This procedure was reversed for the side effect anatomical hierarchy feature. First, for a drug-side effect sample, side effects that were known to be associated with the drug in the training set were identified. Then, the maximum similarity value between the side effect and other side effects was obtained. In particular, if the side effect had a known association with the drug in the training set, the similarity of the side effect itself was excluded.

Accordingly, each pair was represented by seven features.

### 2.3. Machine Learning

Using datasets constructed in the above step, four base machine learning algorithms were applied: random forest (RF), logistic regression (LR), XGBoost (XGB), and naive Bayesian (NB) [28–31]. In the machine learning process, a validation dataset was used to optimize parameters for each algorithm. The test set was used to evaluate the prediction model. In addition, stacking ensemble learning incorporating all these four classifiers as its base classifiers and using a neural network as its meta classifier was applied [32,33].

**Figure 4:**
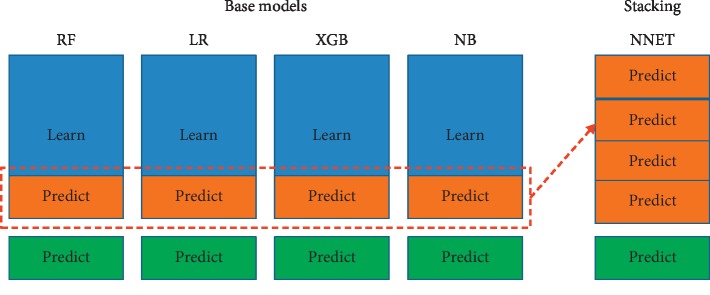
Base classifiers and stacking ensemble learning.

The stacking ensemble learning is illustrated in Figure 4. The training, validation, and test datasets are shown, respectively, in blue, orange, and green colors in the base models. In the stacking step, the predictions from the validation dataset in the base step become the training dataset and are shown in orange color.

### 2.4. Performance Evaluation

In this study, the area under the curve (AUC), precision, recall, F1 score, and specificity were evaluated. Each measurement was averaged across 100 validation datasets and test datasets. True positive (TP) and false positive (FP) indicate the numbers of correctly and falsely predicted positive drug-side effect pairs. True negative (TN) and false negative (FN) represent the numbers of correctly and falsely predicted negative drug-side effect pairs.

(3) $$\begin{array}{c} \text{Precision}=\;\frac{\text{TP}}{\text{TP}+\text{FP}},\\ \text{Recall}=\;\frac{\text{TP}}{\text{TP}+\text{FN}},\\ \text{F}1-\text{score}=\;\frac{2\times \text{Precision}\times \text{Recall}}{\text{Precision}+\text{Recall}},\\ \text{Specificity}=\;\frac{\text{TN}}{\text{TN}+\text{FP}}. \end{array}$$

### 2.5. Confirmation of Drug Candidate Side Effects with FAERS and MedEffect

The average AUC was obtained for each algorithm across validation datasets and test datasets. Considering all the average AUCs, the RF algorithm, which exhibited the best results in the validation and test datasets, was chosen to predict the candidate side effects of drugs, and a classifier was applied. The remaining set, which was not used earlier, was used to predict the candidate associations.

For validation purposes, the drug-side effect associations were extracted from the FDA Adverse Event Reporting System (FAERS), which uploads event lists four times a year (2015.4–2017.9) [34]. FAERS was compiled into 1,829,465 drug-side effect associations for 2,921 drugs and 5,737 side effects. The data from Health Canada's Adverse Drug Reaction Reporting System (MedEffect) was compiled into 2,466 drug-side effect associations for 55 drugs and 902 side effects [35]. FAERS and MedEffect are postmarketing surveillance systems.

There were two types of associations: (1) the associations predicted using the method proposed in this study and (2) the actual associations from FAERS and MedEffect. Two contingency tables were created for FAERS and MedEffect with our predictions as depicted in Table 2. Fisher's exact test (expressed as equation (4)) was applied to check whether the candidate predictions from the proposed method significantly enhanced the existing side effect databases.

(4) $$\begin{array}{c} p=\frac{\left(x+y\right)!\left(z+w\right)!\left(x+z\right)!\left(y+w\right)!}{x!y!z!w!n!}. \end{array}$$

In this equation, the cells of the contingency table are denoted as *x*, *y*, *z*, and *w*; *n* represents the grand total and *p* represents the probability.

## 3. Results and Discussion

Compared with the use of only the target, indication, and chemical properties, the addition of more features improved the capability of predicting the candidate side effects of a drug. The similarities between drugs were evaluated using DDIs from DrugBank (DDIs-D), DDIs from network (DDI-N), SNPs, indications, and the target and chemical properties of drugs. In addition, the similarities between side effects based on side effect anatomical hierarchy were computed. A maximum of seven features for each drug-side effect pair were extracted.

For a drug and a side effect to be considered in the prediction process, a similarity value between that drug and other drugs that had the same side effect was required. Furthermore, only side effects associated with at least five drugs were considered. Accordingly, 638 drugs and 2,117 side effects were used in this study. The number of pairs used in each set is presented in Table 3. The datasets were built by combining a positive set with negative sets. The positive set consisted of known associations from SIDER, whereas the negative sets were randomly generated from the drugs and side effects used in the positive set. The associations included in the positive set were excluded from the negative sets. In total, 100 negative sets were randomly generated, and 100 sets were created by combining positive and negative sets. Then, a set was split into three subsets: training, validation, and test datasets. The ratio of these sets was 8 : 1 : 1, as indicated in Table 3.

### 3.1. Performance Evaluation

Four machine learning algorithms were adopted: NB, RF, LR, and XGB algorithms. First, the performance was tested for a case in which only chemical, indication, and target features (primarily utilized in previous studies) were used. Subsequently, other features were added incrementally. The impact of adding features incrementally is illustrated in Table 4. For each type of feature set, 100 hold-out validation runs were performed, and the resulting AUC scores were averaged.

In Table 4, the “Base” feature comprised the indications, targets, and chemical structures commonly used in previous studies. Next, other features were added to the “Base” feature, with “+” representing the addition of features. For example, “Base + SNPs” indicates that the SNP feature was added to indications, targets, and chemical structures. “SE-AH” denotes the side effect of the anatomical hierarchy feature. Accordingly, 16 different feature combinations were created to demonstrate the improved performance with the inclusion of additional features.

The results presented in Table 4 indicate that the addition of features increased the AUCs for all machine learning algorithms. In the RF model, the AUC increased by approximately 3.5% when new features were considered along with chemical, indication, and target features. Thus, it can be concluded that the addition of new features improved the prediction capability.

Table 5 represents various performance measurements when all features are used. The data indicate that the higher the AUC, the higher the other measurements.

**Figure 5:**
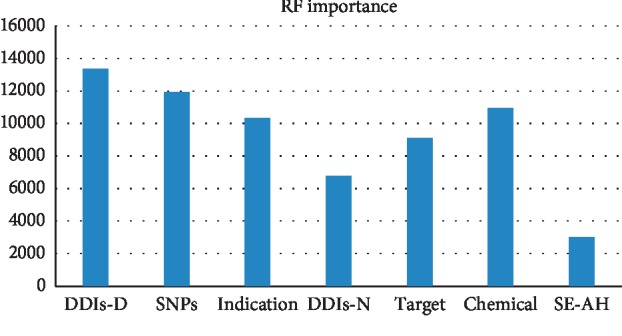
Feature importance of RF.

Table 6 illustrates the relative importance of features when all the features are used. Feature importance signifies the extent of the contribution made by each feature to the performance of the model. We used the varImp function of the caret R package to obtain feature importance [36]. It is evident that new features introduced into our prediction method contribute significantly to the improvement in the overall prediction performance. To elaborate further, we depicted the feature importance of RF in Figure 5. RF showed the best results in Table 4.

As shown in Figure 5, the DDI-D and SNP features play a significant role in the RF model. Therefore, it can be concluded that the incorporation of new features into the method can substantially improve side effect predictions.

### 3.2. Candidate Predictions

The proposed method in this study yielded the best results when the RF model with all the features (DDIs-D, DDIs-N, SNPs, side effect anatomical hierarchy, chemical structures, target, and indication) was used, as depicted in Table 4. Therefore, the classifier that yielded the highest AUCs from the RF model was chosen to predict the candidate side effects of drugs. In the proposed model, 638 drugs and 2,117 side effects were used. An entire set was generated that comprised 1,350,646 pairs of drugs and side effects. The pairs used in the training, validation, and test sets were excluded from the entire pairs. The remaining 1,197,356 pairs were used to predict candidate side effects of drugs. Finally, the candidate predictions for 188,568 novel drug-side effect associations were obtained, as shown in Supplementary [Supplementary-material supplementary-material-1]. A drug could have multiple side effects. The candidate predictions were compared with the existing database for known associations to confirm that the predictions significantly enriched the existing databases. Fisher's test using R was performed in accordance with equation (4) and Table 2, and the results confirmed that the new predictions significantly enriched FAERS (*p* < 2.2*e* − 16 and odd ratio = 2.104912). The contingency table for FAERS and the predictions from the proposed method are reproduced in Table 7. The predictions of the proposed method also significantly enhance MedEffect (*p*=0.001169 and odd ratio = 1.2266).  Table 8 shows the contingency table for MedEffect and the predictions from the proposed method.

### 3.3. Comparison with Previous Studies

We compared our results with those of two previous studies by Zhao et al. because they too treated a drug-side effect pair as a sample, like we did in this study [37, 38]. Two other previous studies used the fingerprint, chemical structure, ATC code, literature association, and target as features to predict side effects and applied diverse machine learning algorithms such as RF, nearest neighbor, dagging, and support vector machine. Zhao et al. adopted similarity and network embedding for feature construction, respectively. They used RF as their preferred method.

In this study, we showed the results of RF in comparison with the results obtained in previous studies [37, 38]. They adopted RF as their final model.

The stacking model was proposed to highlight its slightly improved performance compared to RF. In the stacking ensemble model, we adopted a neural network as a meta classifier, and four models (RF, NB, XGB, and LR) were used as base models. Therefore, we compared our study to previous studies with two proposed methods: stacking and RF.

As seen in Table 9, the two proposed stacking and RF methods yielded better results while predicting the side effects of drugs than the method of Zhao et al. The results show significant differences in three measurements (precision, recall, and F1). Because the F1 score considered both precision and recall, a good F1 score indicates low false positives and low false negatives. It means that our model was less likely to predict false side effects as positive side effects and actual side effects as negative side effects. Therefore, the additional features proposed in this study can be more effective at ascertaining the side effects of drugs than only the features suggested in previous studies.

### 3.4. Case Studies

Of the 188,568 candidate predictions, 12,648 associations were verified by FAERS and 377 associations were verified by MedEffect. To evaluate the practical benefits of the predictive classification models, diverse drugs were sampled according to the following criteria: (1) a recently approved drug with a target-based rational design, (2) a drug for long-term administration, whose side effects required more monitoring, (3) a multitargeted drug showing several differential indications, and (4) a long-established drug whose mechanism was imperfectly understood. They were further examined via a comparison with academic literature and drug discovery material.

First, dasatinib, a small-molecule multikinase inhibitor used to treat cancer, was selected as a recently approved drug with a target-based rational drug design [39]. Consequently, the observational and interventional side effects clinical data on the drug were relatively abundant, and swift data updates for indication expansion were expected. Remarkably, among the 449 predicted side effects of dasatinib, 16 matched the reported data in MedEffect and 194 matched the reported data in FAERS. Accordingly, 43% of the predicted side effects of dasatinib were found in the clinical data. Moreover, literary evidence was found for some predicted side effects present in both MedEffect and FAERS. Angioedema is a serious adverse drug reaction that can be caused by dasatinib [40]. According to a study by Reyes-Habito et al., dasatinib commonly causes skin reactions, such as pruritus, acne, and xerosis [41]. A symptom of “blood alkaline phosphatase increase” can also be caused by dasatinib [42].

Second, sitagliptin, a diabetes therapeutic acting as a selective dipeptidyl peptidase-4 inhibitor, was chosen as a good representative of a drug prescribed for long-term care [43]. Because long-term administration is needed, the side effect monitoring required here is more than that required for short-term administration drugs. Furthermore, sitagliptin also represents a significant new drug class for diabetes. It has a clear mechanism of action and a rational drug design. To develop combination-therapy drugs applicable to a metabolic syndrome caused by sitagliptin and other drugs, researchers need to have a comprehensive understanding of its side effects. Among the 236 predicted side effects of sitagliptin, 134 matched the data reported in FAERS; that is, 56% of the sitagliptin predictions were found in clinical data.

Third, vorinostat, a multitargeting drug was chosen for its widely varying indications. It is used to treat diseases ranging from HIV infection to diverse types of cancers, including nonsmall-cell lung, ovarian, breast, and pancreatic cancers. As vorinostat is a target-based anticancer drug, its side effects are more reasonably traceable than those of other cytotoxic agents. When considering the risk-to-benefit ratio, the side effects of anticancer drugs can be insignificant in comparison with those of other therapeutic drugs. However, recent cancer treatment tends to consider the quality of life as well as the survival rate. Among the 151 predicted side effects of vorinostat, 56 were identified in the FAERS clinical data and 1 was identified in MedEffect. Therefore, 37% of the predictions matched the clinically reported side effects in FAERS, thus proving that the proposed method could be used to effectively identify new side effects from the complete list of drugs with warning black boxes.

Finally, clonidine was chosen as it has an unclear mechanism despite being a long-established drug. Although the available information of its on-target effects was inadequate and could not be used to explain its side effects, the accumulated records of both diverse indications and side effects were sufficient for comparison with the predictions from the proposed method. In total, 563 side effects for clonidine were identified.

To summarize, this study shows that, to a notable extent, predictions can be found in clinically reported data. However, some unprecedented side effects suggested by the predictions of the proposed model will have to be clinically validated in the near future.

## 4. Conclusions

In this study, we proposed a method to deduce the candidate side effects of drugs using the existing knowledge on drugs and side effects. Unlike previous studies that primarily focused on the biological, phenotypic, and chemical characteristics of drugs, in this study, we used an array of information on drugs. The focal point of this study is that properties used in drug repositioning studies can be utilized to predict side effects because the phenotypic expression of a side effect is similar to that of the disease.

By leveraging a variety of data such as SNPs, DDIs, and side effect anatomical hierarchy, the benefits of adopting these data as features were demonstrated; the overall performance was found to be 3.5% better than that obtained by only the target, chemical, and indication features.

The limitation of the proposed method is that it could not identify the candidate side effects of drugs that did not have similarity values in all features. However, the rapid growth of potential information on drugs will ensure the availability of more drugs for further study.

## Figures and Tables

**Table 1:** Information on features used in this study.

Name	Description	Source
Drug-drug interactions from DrugBank (DDIs-D)	Change in the efficacy or toxicity of one drug caused by another drug	DrugBank
Drug-drug interactions from network (DDIs-N)		STRING

Single nucleotide polymorphism (SNP)	Substitution of a single nucleotide that occurs at a specific position in the genome	Fagny et al. [12]

Target protein	Functional biomolecule controlled by drugs	DrugBank

Chemical structure	A molecule represented by a graph with nodes (atoms) and edges (bonds)	DrugBank, PubChem

Indication	Use of drugs for treating particular diseases	TTD, CTD, repoDB

Side effect anatomical hierarchy	Anatomical characteristic of a side effect	Wadhaw et al. [13]

**Table 2:** Contingency table.

Predicted actual	True	False	Row total
True	*x*	*y*	*x* + *y*
False	*z*	*w*	*z* + *w*
Column total	*x* + *z*	*y* + *w*	*n* (=*x* + *y* + *z* + *w*)

**Table 3:** Number of pairs that are used in each set.

	Number of pairs in the positive set	Number of pairs in the negative set	Total number of pairs used in the set
Training set	61,316	61,316	122,632
Validation set	7,664	7,664	15,328
Test set	7,665	7,665	15,330

**Table 4:** Averaged AUCs from our dataset for 100 hold-out validation runs of our machine learning algorithms.

	Validation set				Test set			
Type of feature set	RF	NB	XGB	LR	RF	NB	XGB	LR
All features	**0.9009**	0.8713	0.8917	0.8641	**0.9018**	0.8713	0.8921	0.8642
Base + DDIs-N + SNPs + DDIs-D	0.8964	0.8575	0.8834	0.8501	0.8973	0.8575	0.8835	0.8501
Base + SE-AH + SNPs + DDIs-D	0.8961	0.8710	0.8896	0.8655	0.8970	0.8710	0.8896	0.8656
Base + SE-AH + DDIs-N + DDIs-D	0.8947	0.8645	0.8859	0.8550	0.8959	0.8644	0.8863	0.8550
Base + SNPs + DDIs-N + SE-AH	0.8940	0.8645	0.8868	0.8563	0.8951	0.8644	0.8870	0.8563
Base + SNPs + DDIs-N	0.8905	0.8572	0.8809	0.8519	0.8913	0.8572	0.8809	0.8519
Base + DDIs-D + DDIs-N	0.8901	0.8505	0.8773	0.8400	0.8911	0.8505	0.8773	0.8401
Base + SNPs + DDIs-N	0.8886	0.8496	0.8775	0.8415	0.8897	0.8496	0.8776	0.8414
Base + DDIs-D + SE-AH	0.8879	0.8641	0.8830	0.8563	0.8890	0.8640	0.8830	0.8563
Base + SE-AH + SNPs	0.8874	0.8641	0.8840	0.8574	0.8885	0.8690	0.8840	0.8575
Base + SE-AH + DDIs-N	0.8849	0.8540	0.8783	0.8422	0.8863	0.8539	0.8785	0.8421
Base + DDIs-D	0.8812	0.8502	0.8736	0.8432	0.8820	0.8502	0.8736	0.8431
Base + SNPs	0.8798	0.8493	0.8744	0.8440	0.8810	0.8492	0.8744	0.8440
Base + DDIs-N	0.8788	0.8389	0.8681	0.8281	0.8802	0.8389	0.8683	0.8279
Base + SE-AH	0.8761	0.8535	0.8740	0.8430	0.8774	0.8533	0.8741	0.8429
Base	0.8659	0.8385	0.8634	0.8313	0.8673	0.8385	0.8636	0.8310

**Table 5:** Performance measurements using all features.

	Specificity	Precision	Recall	F1
Validation set				
RF	0.8113	0.8167	0.8407	0.8285
NB	0.7521	0.7655	0.8245	0.7957
LR	0.7913	0.7934	0.8016	0.7975
XGB	0.8161	0.8169	0.8207	0.8188

Test set				
RF	0.8126	0.8178	0.8473	0.8294
NB	0.7514	0.7682	0.8240	0.7951
LR	0.7912	0.7933	0.8014	0.7973
XGB	0.8144	0.7154	0.8196	0.8175

**Table 6:** Representation of feature importance by ranking.

	DDIs-D	SNPs	Indication	DDIs-N	Target	Chemical	SE-AH
RF	1	2	4	6	5	3	7
LR	4	3	5	7	6	2	1
XGB	2	3	5	7	1	4	6
NB	1	2	5	6	4	3	7

**Table 7:** Contingency table for FAERS and predictions from proposed method.

Predictions FAERS	True	False
True	12,648	7,797
False	41,446	53,780

**Table 8:** Contingency table for MedEffect and predictions from proposed method.

Predictions MedEffect	True	False
True	377	1,145
False	4,089	15,233

**Table 9:** Comparison with the method of Zhao et al.

	AUC	Specificity	Precision	Recall	F1
Proposed method (stacking)	**0.9040**	0.8104	0.8174	0.8483	0.8325
Proposed method (RF)	**0.9018**	0.8126	0.8178	0.8473	0.8294
Zhao et al. method (2019)	0.8977	0.880	0.760	0.761	0.761
Zhao et al. method (2018)	0.8492	0.759	0.766	0.791	0.778

## Data Availability

The data used to support the findings of this study are available from the corresponding author upon request.

## Supplementary Materials

Supplementary Materials Supplementary Table 1: 188,568 novel drug-side effect associations.
